# Azelastine–Fluticasone Combination Therapy in Allergic Rhinitis: Current Evidence and Clinical Implications in Children and Adults

**DOI:** 10.3390/ph18111624

**Published:** 2025-10-28

**Authors:** Cristiana Indolfi, Angela Klain, Giulio Dinardo, Carolina Grella, Pierluigi Di Filippo, Ilaria Fatica, Vincenzo Napolano, Fabio Decimo, Michele Miraglia del Giudice

**Affiliations:** Department of Woman, Child and General and Specialized Surgery, University of Campania “Luigi Vanvitelli”, 80138 Naples, Italy; cristianaind@hotmail.com (C.I.); klainangela95@gmail.com (A.K.); pierluigi.difilippo94@gmail.com (P.D.F.); ilaria_0196@hotmail.com (I.F.); vincenzo.napolano@studenti.unicampania.it (V.N.); fabio.decimo@unicampania.it (F.D.); michele.miragliadelgiudice@unicampania.it (M.M.d.G.)

**Keywords:** azelastine–fluticasone (Aze-Flu), allergic rhinitis, pediatric population, intranasal therapy, seasonal allergic rhinitis, perennial allergic rhinitis, efficacy and safety, quality of life, pediatric upper airway disorders

## Abstract

Allergic rhinitis (AR) is a common chronic respiratory disease that significantly impairs the life of children. While a combination intranasal spray of azelastine hydrochloride and fluticasone propionate (Aze-Flu) is an established effective treatment for adults with moderate-to-severe AR, the clinical evidence available in the pediatric population is limited. This review summarizes the current evidence on the efficacy, safety, and impact on Quality of Life (QoL) of Aze-Flu in children. Clinical trials have demonstrated that Aze-Flu provides faster and greater symptom relief in children with AR compared to fluticasone propionate (FP) monotherapy. One randomized controlled trial demonstrated that, although the overall change in the reflective Total Nasal Symptom Score (rTNSS) was not statistically different from the placebo, this was possibly due to rater assessment bias. Children’s symptoms self-assessment showed considerable ameliorations in both nasal and ocular scores. Furthermore, treatment with Aze-Flu has been shown to produce clinically relevant and statistically significant improvements in QoL compared to placebo in children with moderate-to-severe seasonal AR. The safety profile is favorable; a 3-month study confirmed that Aze-Flu is well-tolerated, with an incidence of treatment-related adverse events comparable to that of FP monotherapy. Beyond AR, emerging evidence suggests potential benefits of Aze-Flu in children with adenoid hypertrophy. The available evidence supports Aze-Flu as an effective and well-tolerated therapeutic option for children with moderate-to-severe AR, offering superior and faster symptom control than monotherapy and leading to meaningful improvements in quality of life. Future pediatric trials should incorporate validated, child-specific assessment tools to better capture treatment efficacy.

## 1. Introduction

Allergic rhinitis (AR) is a chronic respiratory disease that is highly prevalent in both adults and children. According to estimates from the Allergic Rhinitis and its Impact on Asthma (ARIA) initiative, AR affects an estimated 10–25% of the Italian population and approximately 15% of individuals in the United States, ranking among the most common chronic allergic conditions worldwide [1,2]. Patients affected by AR experience significantly disabling symptoms (watery rhinorrhea, nasal obstruction, itching of the nose, throat or eyes, frequent sneezing) and, in some cases, comorbidities (atopic dermatitis, allergic conjunctivitis, and asthma), which substantially impact their quality of life (QoL), sleep, daily activities, and consequently work/school performance. AR is one of the clinical entities of the “atopic march” and may precede or accompany the onset of other allergic diseases such as asthma, atopic dermatitis, and food allergy [3]. Allergic asthma is among the most frequent comorbidities in patients with uncontrolled AR [1,4]. Complications of AR include acute or chronic sinusitis, otitis, nasal polyposis, tympanic membrane perforation, Eustachian tube dysfunction, chronic cough, adenoidal hypertrophy, and migraine [5]. These complications may worsen prognosis and represent a major challenge in the chronic management of AR for healthcare providers. Many of them arise from chronic inflammation, the consequent increase in mucus production, and potential bacterial superinfections [5].

Based on symptom frequency and severity, the most recent classification of AR distinguishes two main forms: intermittent allergic rhinitis (IAR) and persistent allergic rhinitis (PAR), which can be further subdivided into mild, moderate, and severe. In the previous classification, AR was divided into two categories: seasonal allergic rhinitis (SAR), when allergen exposure occurred only during a limited period of the year, and perennial allergic rhinitis (PAR), when exposure was present throughout the year [1] (Table 1). Current therapeutic strategies for AR are primarily based on the use of oral second-generation antihistamines and topical intranasal corticosteroids (INCS), which represent the mainstay of treatment for mild to moderate disease. In patients who fail to achieve adequate symptom control with monotherapy, the fixed-dose combination of azelastine hydrochloride (AZE) and fluticasone propionate (FLU) provides an effective step-up option, offering faster and broader relief of nasal and ocular symptoms due to its dual antihistaminic and anti-inflammatory mechanisms of action [1]. However, despite the well-documented efficacy and safety of AZE and FLU in adults, evidence in the pediatric population remains relatively scarce, with few randomized controlled trials (RCTs) specifically addressing this age group and limited long-term data available.

This review of the available literature data aims to summarize the available evidence on the efficacy, safety, and impact of Aze-Flu use on QoL in children and adults.

## 2. Materials and Methods

A literature search was conducted in MEDLINE (via PubMed) and the Cochrane Library to identify studies published in English between January 2015 and September 2025 on the intranasal fixed-dose combination of AZE and fluticasone (FLU) in adults and children with AR. Both interventional (RCTs or non-RCTs) and observational studies conducted in human subjects were considered eligible. The search strategy combined Medical Subject Headings (MeSH) and free-text terms related to azelastine, fluticasone, and allergic rhinitis, using Boolean operators. An example of the MEDLINE syntax is as follows: (“Azelastine”[MeSH Terms] OR “azelastine hydrochloride”) AND (“Fluticasone Propionate”[MeSH Terms] OR “fluticasone”) AND (“Allergic Rhinitis”[MeSH Terms] OR “rhinitis, allergic”) AND (“Child”[MeSH Terms] OR “Adolescent”[MeSH Terms] OR “Adult”[MeSH Terms]). Additional relevant studies were identified through manual reference screening of selected articles and reviews.

Inclusion criteria were defined according to the PICOS framework:

- Population (P): children (≥6 years), adolescents, and adults diagnosed with seasonal or perennial AR;
- Intervention (I): intranasal administration of the fixed-dose combination of AZE and FLU (Aze-Flu);
- Comparator (C): placebo, intranasal corticosteroid monotherapy, or intranasal antihistamine monotherapy;
- Outcomes (O): change in clinical symptom scores (total nasal symptom score (TNSS), total ocular symptom score (TOSS), visual analogue scale (VAS), QoL indices, including Pediatric Rhinitis Quality of Life Questionnaire (PRQLQ), and safety outcomes (treatment-emergent (TEAEs) and treatment-related adverse events (TRAEs));
- Study design (S): RCTs, non-interventional prospective or retrospective studies, and systematic reviews or meta-analyses.

Exclusion criteria included: non-human or preclinical studies; studies not specifying the use of the AZE-FLU combination; articles not in English or lacking full-text availability; studies focused exclusively on other comorbid allergic diseases without reporting AR outcomes.

**Figure 1:**
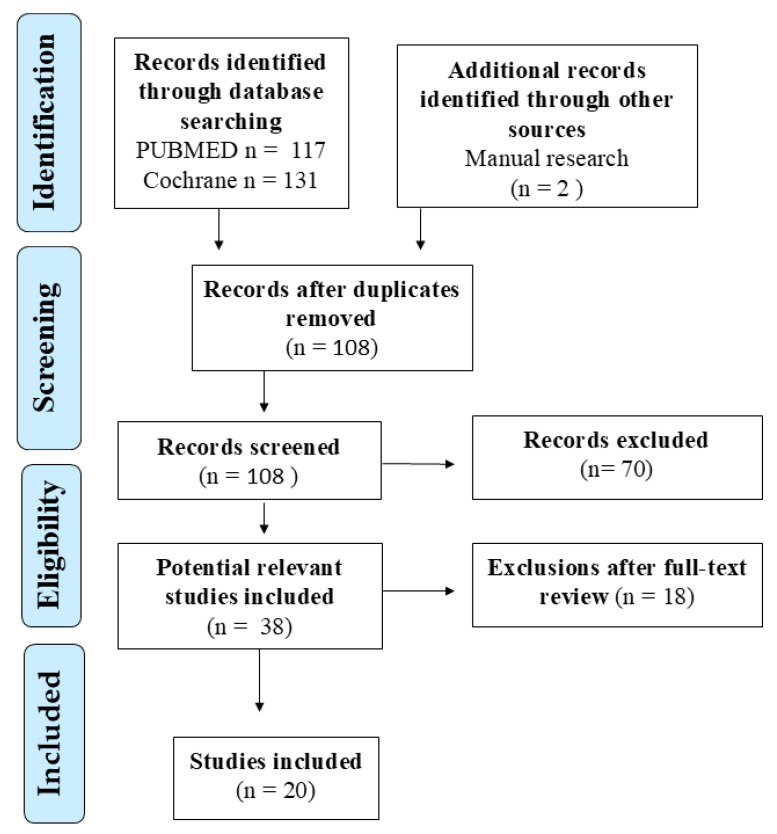
Overview of the Literature Search and Selection Process.

After duplicate removal, all records were independently screened by title and abstract. Full-text articles meeting inclusion criteria were retrieved and assessed in detail by two reviewers (G.D. and C.I.); disagreements were resolved by consensus with a third reviewer (M.M.d.G.) (Figure 1). Extracted data included study design, participant demographics, rhinitis phenotype, treatment duration, efficacy endpoints (rTNSS, rTOSS, VAS, PRQLQ), and safety outcomes. When available, data on comorbidities (e.g., asthma, adenoid hypertrophy), sleep quality, and school performance were also collected.

## 3. Results

AR remains a common and burdensome condition, often insufficiently controlled by standard monotherapies. In the following section, evidence on the use of Aze-Flu combination therapy is presented separately for adults, children, and QoL outcomes, to provide a comprehensive overview of its clinical role (Table 2).

### 3.1. Evidence on Symptom in Adults

AR continues to represent a major clinical challenge, as a significant proportion of patients remain symptomatic despite guideline-based management and the use of standard intranasal therapies [2,6,9,16]. Even when appropriately treated, many adults continue to experience bothersome nasal and ocular symptoms, particularly congestion, rhinorrhea, and sneezing, that impair sleep quality, concentration, and daily functioning [9,10,12,16,17]. Furthermore, persistent or uncontrolled AR has been associated with the exacerbation of several upper and lower airway comorbidities, including asthma, sinusitis, otitis media with effusion, and nasal polyposis, thereby increasing overall disease burden and healthcare utilization [2,6,9,10].

The fixed-dose combination of Aze-Flu was developed to provide dual blockade of histaminergic and inflammatory pathways. By combining the rapid antihistaminic action of azelastine with the potent anti-inflammatory effects of fluticasone, Aze-Flu offers broader symptom control and improved treatment adherence compared with separate agents [2,6,18,19].

Clinical trials and real-world studies consistently confirm the superior efficacy of Aze-Flu compared with either component alone. In a large multicenter, randomized, double-blind study involving 900 adults and adolescents with moderate-to-severe AR, Aze-Flu achieved significantly greater reductions in the 12 h reflective rTNSS and TOSS compared with AZE or FLU monotherapy [6]. A recent meta-analysis including more than 2400 participants further demonstrated that Aze-Flu provided faster and more pronounced improvements in nasal and ocular symptoms than INCS or antihistamines (INAH) alone [17].

Real-world observational studies conducted in Sweden, Romania, Austria, and Ireland corroborate these findings, highlighting both the rapid onset and sustained duration of action [8,10,11,12]. In these studies, most patients achieved well-controlled symptoms, defined as a VAS score < 38 mm, within the first 1–3 days of treatment, regardless of AR phenotype or prior therapy [8,11]. For instance, in a Swedish non-interventional study involving 431 adults with moderate-to-severe AR, the mean VAS score decreased from 67.9 mm at baseline to 32.1 mm after 14 days, corresponding to a 36 mm mean reduction [8]. Similarly, in an Austrian prospective study, VAS scores improved significantly from 53.5 ± 26.3 mm at baseline to 19.6 ± 17.4 mm by day 42 (*p* < 0.0001) [10].

Beyond symptomatic improvement, Aze-Flu has demonstrated measurable anti-inflammatory effects. In an Irish cohort, endoscopic evaluation revealed marked reductions in nasal mucosal edema, erythema, and discharge after 4–6 weeks of therapy [12]. Improvements in olfactory function have also been reported: in a three-month prospective study, 90% of adults with perennial AR regained normal olfactory function following Aze-Flu treatment [7].

Taken together, evidence from RCTs, systematic reviews, and real-world studies supports Aze-Flu as an effective and well-tolerated therapy for moderate-to-severe AR. Its rapid onset, durable efficacy, and favorable safety profile make it a recommended option for adults and adolescents with seasonal or perennial AR, in accordance with ARIA and European treatment guidelines [6,8,10,11,12,20].

### 3.2. Evidence of Clinical Benefits in Children

Clinical evidence on the efficacy and safety of Aze-Flu in children remains limited and largely preliminary compared with adults, as most available studies involved relatively small patient samples and short treatment periods. The approved pediatric age for Aze-Flu differs between regions, being approved for the treatment of SAR in the United States for children aged 6 years and older, while in Europe it is authorized only from 12 years of age due to limited pediatric data [21]. Berger et al. conducted a RCT in children with moderate-to-severe SAR [15]. Participants were randomized to receive either Aze-Flu nasal spray or placebo for 14 days. The primary prespecified endpoint was the change from baseline in the reflective TNSS (rTNSS) over the treatment period, while secondary prespecified endpoints included changes in the reflective TOSS (rTOSS), individual nasal and ocular symptom items, and the PRQLQ. Efficacy analyses were conducted in the intent-to-treat population of children aged 6–11 years (n = 304), whereas all randomized participants aged 4–11 years (n = 348) were included in the safety analysis. In this trial, no statistically significant difference between Aze-Flu and placebo was observed for the primary endpoint (overall change in rTNSS). However, significant improvements over placebo were detected for sneezing and for PRQLQ. The authors hypothesized that the absence of statistical significance for the rTNSS might be related to rater assessment bias, since symptom evaluations were completed either by children or their caregivers. Indeed, subgroup analyses showed that when symptoms were self-rated by children, Aze-Flu provided significantly greater relief across all nasal and ocular symptom domains, whereas this difference was attenuated in caregiver-reported assessments. Nevertheless, the possibility of rater influence should be interpreted cautiously, as the null primary endpoint warrants explicit acknowledgment. The overall risk of bias in this study was considered low-to-moderate, given the adequate randomization and blinding, low dropout rate (<2%), and consistency between intent-to-treat and per-protocol results, although caregiver-by-proxy reporting introduced a potential measurement bias [15]. A more detailed analysis showed that the treatment difference between Aze-Flu and placebo became greater as the proportion of child self-rating increased. When children self-assessed symptoms in > 90% of cases, significant improvements in both rTNSS (*p* < 0.002) and rTOSS (*p* < 0.009) were observed compared with placebo [15]. The main limitations of the study concern the lack of long-term data, the short duration of treatment, and the need for validated pediatric-specific instruments. These findings suggest a potential influence of the rater on reported outcomes, but confirmatory studies are needed to clarify the consistency and robustness of pediatric efficacy signals for Aze-Flu [22].

In another RCT, Berger et al. compared the efficacy of Aze-Flu with FP in children with AR [13]. In this prospective, open-label, active-controlled study, children aged 4–11 years were randomized in a 3:1 ratio to receive either Aze-Flu or FLU nasal spray, both administered twice daily for three months. The primary prespecified endpoint was the change from baseline in the 24 h rTSS, evaluated on a simplified, child-friendly 4-point symptom severity scale ranging from 0 (no symptoms) to 3 (severe symptoms). Although the study was primarily designed to assess long-term safety, efficacy was analyzed secondarily in the population aged 6–11 years (Aze-Flu n = 264; FLU n = 89) using a post hoc mixed-model ANCOVA. Over the 3-month period, Aze-Flu-treated children experienced a mean rTSS reduction in −0.68 points, significantly greater than the −0.54 reduction observed with FLU (difference −0.14; 95% CI −0.28 to −0.01; *p* = 0.04). Superiority was evident from the first day of treatment, particularly within the initial week, and persisted throughout the 90-day study period [13]. A larger proportion of Aze-Flu-treated children achieved symptom-free or only mild symptom status, up to 16 days faster than those receiving FLU, demonstrating faster and more complete control of allergic symptoms. The risk of bias was considered moderate, largely due to the open-label design and absence of predefined inferential efficacy analyses, although the large sample size, multicenter setting, and balanced baseline characteristics support the robustness of the findings. In conclusion, both pivotal studies consistently support the efficacy and safety of Aze-Flu in children aged 6–11 years, demonstrating faster, greater, and more sustained relief of allergic rhinitis symptoms compared with FLU alone [13,15]. The main limitations include the open-label design of one trial, the relatively short follow-up period of three months, and the lack of long-term or QoL outcome assessments [13,15].

Additionally, a 3-month randomized controlled trial involving children aged 4–11 years with AR evaluated the safety and tolerability of Aze-Flu compared with FLU [14]. In this prospective, open-label, multicenter study, participants were randomized in a 3:1 ratio to receive Aze-Flu (n = 304) or FLU (n = 101), administered as one spray per nostril twice daily. The primary prespecified endpoint was the incidence, nature, and severity of TEAEs and TRAEs over 90 days, while secondary safety endpoints included findings from nasal examinations, laboratory parameters, and vital signs. Safety analyses were conducted in all randomized children who received at least one dose of study medication, stratified by age groups (4–5, 6–8, and 9–11 years). The incidence of TRAEs was low and comparable between treatment arms (Aze-Flu 16% vs. FLU 12%). Epistaxis was the most frequently reported event in both groups (9% each), followed by mild headache (3% vs. 1%). All events were mild in intensity and resolved spontaneously, and there were no serious adverse events or clinically relevant changes in laboratory values, vital signs, or nasal examinations. Mucosal findings generally improved over time, and no cases of ulceration or septal perforation were reported. The risk of bias was judged moderate, primarily due to the open-label design and lack of a blinded comparator, although the randomized allocation, large multicenter sample, and high compliance (>96%) strengthen the reliability of the results [14].

Beyond AR, Aze-Flu has also demonstrated clinical efficacy in pediatric patients with uncomplicated adenoid hypertrophy (AH). Bilgili et al. reported significant improvements in nasal obstruction and related symptoms with intranasal Aze-Flu combination therapy [23]. In a subsequent study, the same group showed that Aze-Flu improved Eustachian tube dysfunction and middle ear ventilation in children with AH [24]. These findings suggest that the benefits of Aze-Flu may extend to pediatric conditions characterized by upper airway obstruction beyond AR.

Current pediatric data provide reassurance regarding the pediatric safety of Aze-Flu and support its use as an effective, well-tolerated treatment option in this age group, although evidence remains limited to a few trials with modest sample sizes and short follow-up periods. Differences from adult outcomes may partly reflect developmental factors, such as smaller nasal dimensions, distinct mucociliary function and age-related pharmacokinetic variability, that can influence intranasal drug deposition and treatment response [25,26] underscoring the need for further age-stratified studies to confirm long-term safety and efficacy in this population. A recent systematic review and meta-analysis found INCS to be generally safe in the pediatric population, with only a slight increase in minor local adverse events such as epistaxis [27], however evidence on longer-term use of Aze-Flu in children is scarce.

### 3.3. Evidence on Quality of Life

AR has a significant negative impact on patients’ QoL [28,29]. Impairments in cognitive function, mood, and work or school performance are consistently reported in both pediatric and adult populations [30,31,32]. AR also frequently disrupts sleep, affecting between 50% and 80% of patients, and this sleep impairment is linked to daytime fatigue, mood disturbances such as depression and irritability, and reduced cognitive performance [9,16]. Therefore, effective therapeutic strategies should not only focus on symptom relief but also aim to improve daily functioning and long-term QoL outcomes [33]. Several clinical trials have demonstrated that intranasal Aze-Flu produces clinically meaningful improvements in health-related QoL measures compared with monotherapy [6,16,34].

A recent Chinese RCT enrolled 898 adults with moderate-to-sever AR. Participants were randomly assigned to receive Aze-Flu, AZE or FLU nasal spray for 14 days [23]. QoL improvement was observed in all treatment groups, but was more pronounced in the Aze-Flu group [23]. Similarly, a 2024 multicenter prospective observational study by Marth et al. demonstrated significant improvements in patient-assessed sleep quality after 35 days of Aze-Flu treatment in patients with moderate-to-severe PAR [29].

Another non-interventional study in 53 adults with moderate-to-severe PAR reported sustained improvement in sleep quality throughout the observation period [30].

Additionally, Berger et al. reported clinically relevant and statistically significant improvements in QoL in children with moderate-to-severe SAR treated with Aze-Flu compared with placebo during a 14-day RCT [9]. In this pediatric trial, QoL was assessed using the PRQLQ, which represents the age-adapted and validated version of the widely used adult Rhinoconjunctivitis Quality of Life Questionnaire (RQLQ). Both instruments share comparable domains, such as nasal and ocular symptoms, activity limitations and practical problems, allowing indirect comparison of QoL outcomes between pediatric and adult populations [31]. Nevertheless, pediatric-specific evidence remains limited, and additional trials focusing on QoL outcomes in children are warranted.

## 4. Discussion

### 4.1. Therapeutic Management of Allergic Rhinitis in Children

AR in children represents a significant public health challenge due to its high prevalence, chronic nature, and broad impact on daily functioning [35,36,37,38]. Although therapeutic options such as INCS and INAH alone remain the cornerstone of management, a considerable proportion of pediatric patients continue to experience poorly controlled symptoms [39,40]. This gap underscores the need for more effective treatment strategies, particularly for those with moderate-to-severe disease.

The treatment of AR involves two levels of intervention: environmental prophylaxis and pharmacological therapy. Environmental prophylaxis consists of limiting exposure to inhalant allergens, the main triggers of nasal eosinophilic inflammatory responses [1]. A detailed clinical history can help identify the inhalant allergens responsible for the symptoms [41]. The most common indoor allergens include dust mites, molds, and domestic animals such as dogs and cats. Removal of pillows, curtains, carpets, sofas, and upholstered furniture, as well as the use of dust-mite–proof mattress covers, represent possible interventions for patients allergic to dust mites [42]. Conversely, patients allergic to pollens should keep windows closed during peak pollen season and avoid prolonged stays in areas with high allergen concentrations.

Current recommendations for pharmacological therapy consist of an algorithmic approach, titrating medication based on disease severity [1,28]. This strategy may allow discontinuation of treatment once the pollen season has ended in SAR patients; however, PAR patients may require year-round therapy [2,28]. Topical therapy has the advantage of being administered directly at the site of disease, increasing local drug concentration and efficacy, while reducing systemic side effects due to low absorption through the nasal mucosa. These benefits make intranasal topical drugs the first-line treatment for AR [2].

In addition to pharmacological therapy, adequate nasal hygiene is recommended, achieved through frequent nasal irrigations with isotonic saline or hypertonic saline solutions to prevent mucus accumulation and potential superinfections. INCS inhibit T-cell activation, reduce levels of inflammatory cytokines (interleukin (IL) 2, IL-4, IL-5), and block recruitment of mast cells, eosinophils, and basophils in the mucosa. INAH stabilize histamine receptors, reducing mediator release in the Th2 inflammatory response. Both INCS and INAH can be used as maintenance therapies, having demonstrated efficacy in reducing inflammatory processes [17].

### 4.2. Evidence and Clinical Role of Azelastine–Fluticasone Combination

In IAR, first-line therapy consists of antihistamines, with no clear differences between oral and intranasal formulations. In PAR, INCS are already recommended for mild cases, while the combination of INCS and INAH is reserved for moderate-to-severe cases with a VAS score ≥ 5, particularly when monotherapy has proven insufficient. This combination is also suggested for patients with IAR or PAR and a VAS < 5 when moderate-to-severe nasal congestion is present [2,43]. Systemic steroid therapy is not considered a valid option, due to limited efficacy and a high risk of side effects. Pseudoephedrine and other intranasal vasoconstrictors may provide rapid but temporary symptom relief, especially for nasal congestion. However, since they do not act on the underlying inflammation, they are not recommended for maintenance therapy [17], especially in children. Nasal sprays containing AZE and FLU are among the most widely prescribed intranasal medications for AR worldwide [1,28,44]. AZE is a second-generation H1-receptor antagonist, approved by AIFA for pediatric use starting at 6 years of age, available as a nasal spray and as eye drops for allergic conjunctivitis. FP is a moderately potent inhaled corticosteroid with a broad spectrum of indications for upper and lower respiratory tract diseases. In some moderate-to-severe cases, monotherapy with INCS or INAH may not sufficiently control nasal and ocular symptoms of AR. The introduction of the fixed-dose combination Aze-Flu, which combines AZE and FLU in a single intranasal device, represents an additional therapeutic option for maintenance treatment of AR in adults and adolescents over 6 years of age who are inadequately controlled with INCS monotherapy. Acting on different pathogenic mechanisms of the T helper type 2 cell (Th2) response, INCS and INAH exert a synergistic effect in reducing inflammation and symptoms. Furthermore, the use of a single device to deliver this combination appears to enhance drug efficacy and improve patient adherence to treatment.

**Figure 2:**
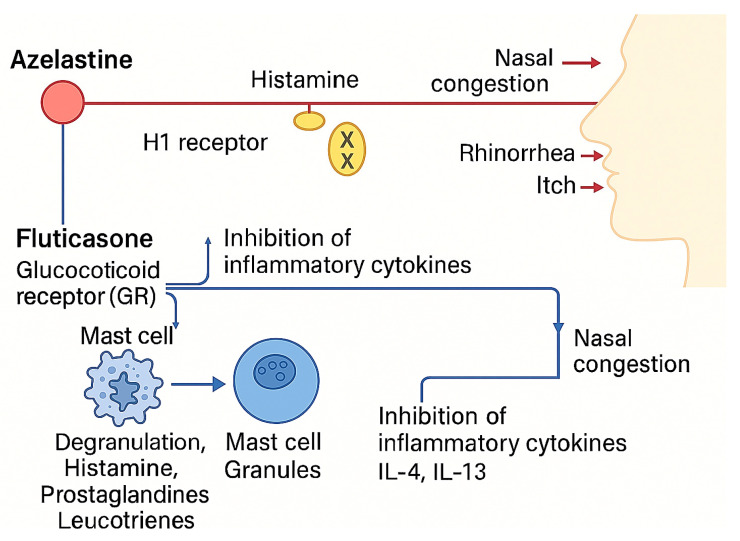
Mechanisms of action of azelastine and fluticasone in AR. Azelastine, by blocking H1 receptors, inhibits mast cell degranulation and reduces the production of pro-inflammatory cytokines, relieving itching, sneezing, rhinorrhea, and nasal congestion; fluticasone, by binding to intracellular glucocorticoid receptors, decreases the production of pro-inflammatory cytokines and leukocyte infiltration in nasal tissues, improving nasal obstruction.

Although available studies indicate that the intranasal Aze-Flu combination is both effective and well tolerated, its positioning in pediatric AR management is still not commensurate with the level of evidence, highlighting the need for further dedicated research (Figure 2).

A key issue in the current literature is the relative paucity of data synthesizing the evidence on combination therapies [45,46,47]. Although monotherapies have been extensively investigated and their efficacy well established through several systematic reviews and meta-analyses, evidence regarding intranasal fixed-dose combinations remains comparatively limited [48,49]. Recent comprehensive syntheses by Sousa-Pinto et al. have substantially expanded this knowledge base, demonstrating that the Aze-Flu combination achieves the greatest overall efficacy among available intranasal treatments for AR, with clinically meaningful reductions in TNSS, TOSS, and RQLQ and no increase in treatment-related adverse events [47,48]. Nevertheless, these meta-analyses primarily included adult or mixed-age cohorts, and pediatric data were often limited or analyzed only as small subgroups [48,49,50,51,52]. Consequently, age-specific conclusions remain scarce, underscoring the need for focused reviews. As a result, clinicians have limited consolidated guidance on when and how to incorporate Aze-Flu into pediatric treatment algorithms. This scarcity of comprehensive syntheses represents an important knowledge gap and highlights the relevance of reviews that bring together the scattered evidence available to date.

From a clinical perspective, combination therapy offers several theoretical and practical advantages. By targeting multiple pathophysiological mechanisms, anti-inflammatory effects from corticosteroids and antihistaminic activity from AZE, the dual approach may provide more complete and faster relief than monotherapy. In pediatrics, where symptom control is closely linked to school performance, sleep quality, and psychosocial well-being, these advantages could be particularly meaningful [51]. Furthermore, using a single intranasal spray to deliver two active agents simplifies treatment regimens, potentially improving adherence compared with the concurrent use of separate medications [52,53]. Improved adherence is especially important in children and adolescents, where the burden of daily therapy and parental supervision often limits treatment effectiveness [54].

Several limitations of the existing evidence base must be acknowledged. First, most pediatric trials are short in duration, usually spanning a few weeks, and thus provide limited insight into long-term safety and sustained effectiveness. Given that AR is often a chronic condition with seasonal or perennial triggers, assessing the durability of treatment benefits and the risk of cumulative adverse effects is essential. Second, outcome measurement in children remains a challenge. Many studies rely on tools validated in adults or on caregiver-reported outcomes, which may not accurately capture the child’s subjective experience. The observation that treatment effects are more evident when children self-report symptoms underscores the need for validated, child-friendly assessment instruments. Without these tools, the true magnitude of benefit may be underestimated or inconsistently reported.

### 4.3. Future Directions, Safety, and Regulatory Considerations

Economic considerations also deserve greater attention. Combination therapy is associated with higher direct costs compared with corticosteroid monotherapy, which could limit accessibility in healthcare systems with constrained resources. However, the broader perspective of cost-effectiveness suggests potential advantages: fewer missed school days, reduced parental work absenteeism, improved daily functioning, and possibly fewer physician visits or additional medications. Robust health-economic analyses in pediatric populations are lacking but would be valuable in defining the positioning of Aze-Flu within therapeutic algorithms.

Moreover, allergen immunotherapy (AIT) represents the only etiopathogenic treatment capable of modifying the natural course of AR by inducing long-term immune tolerance to causal allergens. Its early initiation, particularly in monosensitized children and young adults, is strongly recommended as an adjunct to symptomatic therapy, since it can prevent disease progression, reduce the risk of new sensitizations, and potentially decrease the development of asthma. Both subcutaneous (SCIT) and sublingual (SLIT) routes have demonstrated sustained efficacy and safety in this population, with several RCTs supporting significant symptom and medication score reductions compared to placebo. Nevertheless, AIT is not always a feasible therapeutic route in daily clinical practice. Many patients exhibit polysensitization to multiple allergens, which complicates the identification of a clinically dominant allergen and may limit the effectiveness of single-allergen formulations. In addition, the high cost, the need for long-term adherence, and the requirement for specialist supervision can restrict its applicability, especially in moderate-to-severe forms of AR where polytherapy is often necessary. Despite these practical limitations, AIT remains an essential component of comprehensive AR management and should be considered as early as possible, ideally after 5 years of age in appropriately selected patients. Its inclusion in treatment algorithms reflects the growing emphasis on disease-modifying strategies rather than purely symptomatic control. Therefore, while accessibility and patient selection remain critical challenges, the therapeutic potential and long-term benefits of AIT warrant a more prominent discussion within the management framework of pediatric and young adult with AR [55].

Pediatric safety and regulatory aspects of Aze-Flu warrant careful consideration. Evidence from controlled studies indicates that, in children and adolescents, Aze-Flu exhibits a generally favorable short-term safety profile, with TRAEs occurring at rates comparable to those observed with fluticasone monotherapy [13,14,56]. The most commonly reported events include mild epistaxis, local nasal irritation, and transient dysgeusia, all of which are typically self-limiting and rarely lead to treatment discontinuation. Importantly, no clinically significant systemic corticosteroid-related effects have been identified in pediatric studies, and hypothalamic–pituitary–adrenal axis suppression has not been demonstrated. However, long-term safety data in younger age groups remain scarce.

From a regulatory perspective, current product labeling restricts the use of Aze-Flu to patients aged 12 years and older in most regions, including approvals by the European Medicines Agency (EMA). Only in the United States Aze-Flu is approved for the treatment of SAR for children aged 6 years. Consequently, its use in younger children remains generally off-label and should not be generalized or recommended without appropriate regulatory revision supported by high-quality pediatric RCTs. Given the physiological and metabolic differences in younger populations, extrapolation from adult or adolescent data must be approached with caution. Clinicians should remain aware of potential local effects such as epistaxis or irritation and monitor for rare systemic corticosteroid outcomes (Table 3). Broadening the use of Aze-Flu to younger children may provide clinical value by enhancing symptom control and by potentially improving common comorbid conditions through more effective management of upper airway inflammation [57,58]. The potential role of Aze-Flu beyond AR, such as in AH or Eustachian tube dysfunction, further broadens its clinical relevance. These conditions share overlapping inflammatory mechanisms and contribute significantly to pediatric morbidity [23,24]. Future RCTs will further clarify the long-term efficacy and safety of Aze-Flu in the pediatric population.

## 5. Conclusions

The combination of Aze-Flu represents an evidence-based, step-up option for children with moderate-to-severe AR not adequately controlled by monotherapy, providing faster and more complete symptom control compared to monotherapy. At present in Europe, this association is approved for patients aged 6 years and older, which limits its routine application in younger children despite encouraging evidence from clinical trials. From a clinical standpoint, Aze-Flu may help reduce disease burden, improve adherence by simplifying treatment regimens, and potentially lower indirect costs related to school absenteeism, impaired daily functioning, and recurrent physician visits. The topical literature, however, provides only a fragmented picture, with relatively few pediatric-focused studies and almost no comprehensive syntheses of combination therapy outcomes. Future multicenter trials, involving a larger pediatric population, and long-term safety studies in children under 12 years of age, could represent a way to expand the current European regulatory approval, and better define the positioning of Aze-Flu within therapeutic algorithms. Taken together, these data support Aze-Flu as a valuable option for patients with insufficient control on standard therapy, reinforcing its role as an important advancement in the management of pediatric AR.

## Figures and Tables

**Table 1:** Classification of allergic rhinitis based on symptom frequency and severity, with recommended therapies for each level of severity. Includes first-, second-, and third-line treatment options based on therapeutic response [1].

AR Type	Symptom Frequency	Symptom Severity	Recommended Therapy	Notes
Intermittent	<4 days/week or <4 weeks	Mild: no sleep disturbance, no impairment of daily activities, work/school, sport/socialization	Second-generation oral antihistamines or intranasal corticosteroids (INCS)	Effective in mild or intermittent cases
Persistent	≥ 4 days/week or ≥ 4 weeks	Moderate-severe: sleep disturbance, impairment of daily activities, work/school, sport/socialization	INCS (first-line), or INCS + intranasal antihistamine, or short-term decongestants	First-line treatment highly effective for persistent or moderate-severe symptoms; combination therapies if monotherapy insufficient; rapid relief but decongestants not for chronic use
Severe/Refractory	Any frequency/severity	Severe, refractory symptoms	Additional combination therapies; consider intranasal cromolyn	For patients not responding to first- or second-line therapy

**Table 2:** Characteristics and Main Findings of Studies Evaluating Azelastine–Fluticasone Combination in Adult and Pediatric Populations.

	Study (Year, Ref.)	Study Design	Study Population and Design Features	Demographics	Baseline Characteristics	Main Findings
Adults	Zhou 2023 [6]	Multicenter, randomized, double-blind, active controlled prospective clinical study	898 male and female patients ≥ 12 years of age with moderate-to-severe rhinitis or rhino conjunctivitis with a minimum 2-year PAR history. Participants were randomly assigned to receive either Aze-Flu nasal spray or AZE nasal spray or FLU nasal spray for 14 days	The mean (SD) age of the patients was 35.8 (11.83) years.	The mean (SD) baseline combined rTNSS was 17.18 (3.35) and the mean (SD) baseline combined rTOSS was 7.90 (4.94).	Aze-Flu treatment demonstrated a significant superiority when compared with the AZE or the FLU group in reduction in rTNSS or rTOSS scores.
	Klimek 2017 [7]	Prospective, multicenter, observational study	47 patients (≥18 years) with mild, moderate or severe PER Patients had to apply Aze-Flu (1 spray per nostril bid) over a period of 3 months	Average age 31 years (SD, 7.8 years)	VAS: 84.3 (±10.9) mm. Severe PER patients (n = 14) had an average TDI score of 20.0 points. Moderate PER patients (n = 27) had an average TDI score of 24.4 points	Aze-Flu significantly improved olfaction (Severe PER; TDI: 20.0 → 34.7 at 1 mo → 37.1 at 3 mo; *p* < 0.001; moderate PER, TDI 24.4 → 34.0 at 1 mo → 36.8 at 3 mo; *p* < 0.001) and symptom severity (VAS: 84.3 → 32.4 (±7.6) at 1 mo → 26.2 (±7.2) at 3 mo; *p* < 0.001).
	Stjärne 2019 [8]	Multicenter, prospective non interventional study	431 patients aged ≥12 years with ARIA-defined moderate to severe SAR or PAR for whom monotherapy with either an intranasal antihistamine or glucocorticoid was not considered sufficient. Participants were instructed to receive Aze-Flu, one spray in each nostril twice daily, for 14 days.	Most participants were adults aged 18 to 65 years (87.5%); mean age was 42.0 (15.6) years	About three-quarters of the patients had SAR (either SAR alone or SAR + PAR), with only 13.9% diagnosed with PAR only. Patients assessed symptom severity using a VAS from 0 to 100 mm. 367 patients (85.2%) had a baseline VAS score ≥ 50 mm. Nasal congestion was patients’ most frequent predominant symptom (63.1%).	Aze-Flu led to rapid AR symptom relief from the first day of treatment, which was maintained for the duration of the study, assessed using a VAS, the MACVIA-ARIA–endorsed language of AR control. Aze-Flu was shown to reduce mean (SD) VAS score from 67.9 (16.1) mm at baseline (n = 391) to 32.1 (22.8) mm on the last day (n = 372), a reduction of 36.1 (24.0) mm (n = 370).
	Scadding 2017 [9]	Multicenter, non-interventional study	193 patients (≥12 years) with ARIA-defined moderate-to-severe SAR or PAR and acute symptoms, for whom monotherapy with either intranasal antihistamine or glucocorticoid were not considered sufficient	The mean (SD) age of the study population was 37.6 (16.9) years	Most patients had SAR either alone (10.4%) or in combination with PAR (35.2%), but many had AR of unknown origin (35.8%). Patients reported troublesome symptoms (78.2%) and sleep disturbance (64.8%), with nasal congestion considered the most bothersome (54.4%) and ocular symptoms reported by 68.4% of patients.	Aze-Flu was mainly prescribed due to insufficient prior therapy, highlighting the burden of uncontrolled AR. It has been shown to effectively improve symptoms, outperforming intranasal corticosteroids or antihistamine monotherapy and enhancing disease control
	Marth 2024 [10]	Multicenter, prospective, non-interventional, observational study Study	214 participants (≥12 years) with moderate-to-severe PER, who were prescribed Aze-Flu.	Mean age 39.5 ± 16.8 years (range 12–82 years); mean duration of history of AR was 9.8 ± 10.1 years.	Baseline mean VAS symptom score: 53.5 ± 26.3 mm; ≥2 AR medications were used by 55.1% of patients in the past; 25.7% had current or prior immunotherapy. The percentage of patients with very good/good sleep quality on day 0 was 27.5% (58/211)	Aze-Flu provided rapid and significant symptom relief (baseline VAS 53.5 ± 26.3 mm → 47.7 ± 25.6 mm on day 1 → 25.3 ± 21.0 mm on day 28 → 19.6 ± 17.4 mm on day 42; all *p* < 0.0001), effective across ages, sexes, severities, and phenotypes. The percentage of patients with very good/good sleep quality increased to 87.2% (95/109) on day 42
	Agache 2018 [11]	Prospective, multicenter, non-interventional study	253 patients (≥12 years) with moderate-to-severe SAR or PAR were prescribed MP-AzeFlu and instructed to use it for 14 days.	90.9% of patients were adults aged 18–65 years (n = 230)	249 patients (98.4%) had a baseline VAS score > 50 mm. Mean (SD) VAS score: 78.4 (15.1) mm. Mean (SD) duration of AR: 21.6 (11.5) years. Predominant AR symptom was nasal congestion (n = 151; 59.7%).	Aze-Flu induced rapid VAS score reduction [VAS: 78.4 mm (SD 15.1) at day 0 → 14.7 mm (SD 15.1, *p* < 0.0001) at last day of treatment. Perception of ‘well-controlled’ symptoms corresponded to a VAS score ≤ 38 mm. Patients who achieved this cutoff: 10.9% at Day 1 → 36.8% at Day 3 → 70.9% at Day 7 → 83.4% at study end.
	Kaulsay 2018 [12]	Prospective, non-interventional study.	53 patients (≥12 years) with moderate-to-severe PER who were prescribed Aze-Flu.	Mean age 31.2 ± 15.2 years (median, 31 years; range, 12–69 years).	VAS symptom score: 73.4 mm (SD 20.3). Total endoscopy score: 7.5 mm (SD 3.1). Percentage of patients with very good or good sleep quality: 25.0% (13 of 52 patients)	Aze-Flu induced rapid VAS score reduction [VAS: 73.4 mm (SD 20.3) at day 0 → 31.5 mm (SD 25.0, *p* < 0.0001) at day 28 → 28.1 mm (SD 24.1, *p* < 0.0001) at day 42], a 45.3 mm reduction. Endoscopy score improved from baseline (7.5) to 3.5 mm (SD, 2.5) at day 28. Improved sleep quality was also observed.
Children	Berger 2016 [13]	Prospective, randomized, active-controlled, parallel-group, open-label safety and efficacy study.	405 children aged ≥4 to <12 years with AR; randomized in 3:1 ratio to either Aze-Flu nasal spray (n = 304) or FP nasal spray (n = 101).	48.5% of patients were aged ≥6 to <9 years and 51.5% were aged ≥9 to <12 years in the Aze-Flu group.	Similar baseline characteristics were observed in the MP-Aze-Flu and FP groups. The proportion of children with concomitant asthma was 4.86% and 4.90% in the Aze-Flu and FP groups, respectively.	Aze-Flu provided significantly greater symptom relief than FP, with a mean TSS reduction in −0.68 points compared to −0.54 with FP (difference −0.14; 95% CI: −0.28 to −0.01; *p* = 0.0410). Around 80% of Aze-Flu–treated children achieved symptom-free or mild-symptom status within 1 month-approximately 16 days faster than FP.
	Berger 2018 [14]	Multicenter, randomized, open-label, active-controlled safety trial	405 children, aged 4–11 years, with AR. Patients were randomized in a 3:1 ratio to Aze-Flu (n = 304) or FP (n = 101), and to one of three age groups: ≥4 to <6 years (12.6%), ≥6 to <9 years (42.7%), and ≥9 to <12 years (44.7%).	Patients with history of AR (SAR or PAR), who could benefit from treatment with Aze-Flu	Aze-Flu group: mean TSS score ± SD (1.72 ± 0.76); mean PRQLQ score ± SD (1.84 ± 1.05) FP group: mean TSS score ± SD (1.77 ± 0.73); mean PRQLQ score ± SD (1.88 ± 1.15)	The percentage of subjects with ≥ 1 TEAE and ≥ 1 TRAE over 3 months was similar in the Aze-Flu (41% and 16%) and FP groups (37% and 12%). Aze-Flu was safe and well tolerated after 3 months’ continuous use in children with AR
	Berger 2016 [15]	Randomized, double-blind, multicenter placebo-controlled, parallel-group,14-day trial	348 children (4–11 years) with moderate/severe SAR. After a 3–7 day single-blind placebo lead-in, children stratified by age were randomized to receive either Aze-Flu nasal spray or placebo for 14 days.		A history of SAR and positive skin prick test showing hypersensitivity to prevailing pollen, with a rTNSS ≥ 6 and reflective congestion score ≥ 2 on the first day of placebo lead-in.	Aze-Flu showed statistically significant and clinically relevant improvement in PRQLQ scores vs. placebo (–0.29; 95% CI –0.55 to –0.03; *p* < 0.027). When children self-assessed symptoms > 90% of the time, significant improvements in rTNSS (*p* < 0.002) and rTOSS (*p* < 0.009) were observed compared to placebo.
	Krishnakumar 2022 [16]	Prospective comparative non-randomized study	240 patients (≥4 years) with moderate to severe allergic rhinitis. Participants divided into group A (120 patients) receiving fluticasone nasal spray, and group B (120 patients) receiving azelastine nasal spray, both for a period of three months along with an oral antihistamine for one week	Median age of patients in group A was 32.5 (23) and that in group B was 31.5 (20.75)	Baseline TNSS medians: Group A 10 (IQR 4); group B 9 (IQR 4) Baseline median (IQR) nasal congestion score of group A and B was 3 (1)	Both medications significantly reduced TNSS (to median 1) after 3 months (*p* < 0.001), with no significant difference between groups (*p* = 0.56–0.06); and significantly reduced nasal congestion score (to median 0, *p* < 0.001), with no significant difference between groups (*p* = 0.31–0.14)

**Table 3:** Practical Considerations for Aze-Flu Therapy in Children.

Aspect	Key Points for Clinical Practice
When to use	Consider Aze-Flu in children with moderate-to-severe allergic rhinitis who remain symptomatic despite adequate intranasal corticosteroid (INCS) monotherapy, especially in cases with persistent nasal congestion or ocular symptoms.
Assessment	Use simple, validated child-reported tools (e.g., Visual Analogue Scale, Pediatric Rhinoconjunctivitis Quality of Life Questionnaire) to monitor symptom control and quality-of-life improvement.
Adherence	One-device therapy improves convenience and compliance. Demonstrate proper spray technique and encourage consistent daily use.
Safety	Monitor for local adverse effects (epistaxis, nasal irritation) and track growth during prolonged treatment.
Regulatory note	Approved for use from 6 years of age in the US and from 12 years in Europe; follow local labeling and regulatory guidance before prescribing.

## Data Availability

No new data were created or analyzed in this study. Data sharing is not applicable to this article.
